# Phenotypic responses of foxtail millet (*Setaria italica*) genotypes to phosphate supply under greenhouse and natural field conditions

**DOI:** 10.1371/journal.pone.0233896

**Published:** 2020-06-03

**Authors:** S. Antony Ceasar, M. Ramakrishnan, K. K. Vinod, G. Victor Roch, Hari D. Upadhyaya, Alison Baker, S. Ignacimuthu

**Affiliations:** 1 Division of Plant Biotechnology, Entomology Research Institute, Loyola College, Chennai, India; 2 Centre for Plant Sciences, School of Molecular and Cellular Biology, Faculty of Biological Sciences, University of Leeds, Leeds, United Kingdom; 3 State Key Laboratory of Subtropical Silviculture, Zhejiang A&F University, Hangzhou, Zhejiang, China; 4 Division of Genetics, ICAR—Indian Agricultural Research Institute, New Delhi, India; 5 The University of Georgia, Athens, GA, United States of America; 6 Xavier Research Foundation, St. Xavier’s College, Palayamkottai, India; University of Hyderabad, INDIA

## Abstract

Food insecurity is a looming threat for the burgeoning world population. Phosphorus (P), which is absorbed from soil as inorganic phosphate (Pi), is an essential macronutrient for the growth of all agricultural crops. This study reports phenotype analysis for P responses in natural field and greenhouse conditions, using 54 genotypes of foxtail millet (*Setaria italica*) representing wide geographic origins. The genotype responses were assessed in natural field conditions in two different seasons (monsoon and summer) under Pi-fertilized (P+) and unfertilized (P-) soil for eight above-ground traits. Enormous variations were seen among the genotypes in phenotypic responses for all the measured parameters under low P stress conditions. Variations were significant for plant height, leaf number and length, tillering ability and seed yield traits. Genotypes ISe 1234 and ISe 1541 were P+ responders, and the genotypes ISe 1181, ISe 1655, ISe 783 and ISe 1892 showed tolerance to low P for total seed yield. Genotypes that performed well under P- conditions were almost as productive as genotypes that performed well under P+ conditions suggesting some genotypes are well adapted to nutrient-poor soils. In the greenhouse, most of the genotypes produced changes in root architecture that are characteristic of P- stress, but to differing degrees. Significant variation was seen in root hair density and root hair number and in fresh and dry weight of shoot and root under P- stress. However, there was not much difference in the shoot and root total P and Pi levels of five selected high and low responding genotypes. We noticed contrasting responses in the greenhouse and natural field experiments for most of these genotypes. The leads from the study form the basis for breeding and improvement of foxtail millet for better Pi-use efficiency.

## 1 Introduction

As a component of biomolecules such as nucleotides, phospholipids and phosphorylated intermediates of several processes including photosynthesis and respiration, phosphorus (P) plays several important functions in living systems. P is an irreplaceable, essential element for crop production. Plants take up P from the soil in the form of inorganic phosphate (Pi) and the process is affected by several external factors such as soil pH, microbial activity, cationic abundance and the presence of mycorrhizal fungi [1]. Even where soils are rich, P uptake remains a limiting factor for plant growth, due to chemical fixation in organic or inorganic form. Limiting Pi in soil leads to poor soil health and can be a major impediment for crop growth. Pi-deficiency significantly hampers crop production, affecting 5.7 billion hectares of land worldwide [2]. To sustain crop production in continuous cropping systems, replacement of depleted nutrients is carried out through the external application of fertilizers. However, in the case of Pi, natural reserves of rock phosphate, the prime source of inorganic phosphate fertilizers, may only last for the next 200 years [3, 4]. Therefore, the sustainable use of phosphate is an important requirement.

The low Pi use efficiency (PUE) of modern cultivars (around 20%) poses a problem in intensive cropping systems, where phosphatic fertilizer input requirement is high. Most unutilised Pi inputs are either fixed in the soil and/or are leached into water bodies [5]. Widespread increase of Pi deficiency in continuous cropping systems, in spite of external inputs, is a major concern in modern agricultural systems. Input reduction under such circumstances will only exacerbate Pi starvation in crop plants [6]. Thus, there is an urgent need to improve the PUE of crops for sustainable agriculture.

PUE in plants has two components: Pi acquisition efficiency (PAE) or the extent to which plants acquire Pi from the soil, and Pi utilization efficiency (PUtE) or the efficient use of internal P resources. Since both components are complementary, simultaneous improvement of PAE and PUtE is required for sustainable crop production with reduced reliance on external Pi supplementation [7, 8]. Several approaches have been adopted to improve crop PAE and PUtE. These include transgenic- and breeding-based approaches and both approaches have been reviewed by Baker et al. [9] and Lopez-Arredondo et al. [10]. Extensive phenotyping studies have been performed to identify genotypes with greater PUtE and to map associated quantitative trait loci (QTL) for use in breeding programs and these have been reviewed by Wiel et al. [11]. Most of these studies focus on P starvation tolerance because the improvement of P acquisition from soil and utilisation are the prime objectives of breeding for PUE in crops. Under P starvation, P homeostasis is sustained through adaptive mechanisms such as improved nutrient foraging in the rhizosphere through root architectural manifestations that include root proliferation and deeper penetration [12].

Root architectural modification improves topsoil foraging and, therefore, it appears to be particularly important to improve the PAE in P-deficient soils [12]. Root architectural adaptations in response to Pi starvation have been documented in several plant species. In *Arabidopsis*, Pi starvation is reported to accelerate the development of a root system that is highly branched by the development of lateral roots and root hairs [2]. In several other crops such as rice, maize, common bean, white lupin, tomato, and black mustard, root modifications such as primary root length reduction, improved branching, increased lateral root length, enhancement in number of lateral roots and root hair proliferation were reported as Pi starvation responses [2, 13–18]. In previous studies, seedlings of finger millet, foxtail millet, pearl millet, kodo millet, little millet, proso millet, and barnyard millet grown under low Pi condition had altered root morphology and phosphate uptake activity [19, 20]. Roots play a major role in changing the rhizosphere by rhizochemical reactions due to root exudates and due to increased microfloral symbiosis that aid P solubilization and Pi uptake. Root level adaptation augments the innate ability of plants to uptake Pi from the soil, thereby making it a potential trait to target improvement in PAE [21–23].

Besides root level modifications, aerial parts of cereal crops also show key phenotypic modifications in response to low P starvation. These include stunted shoot growth, dark green leaf and reduced yield in oat [24], increased primary root length and reduced photosynthesis in rice [25, 26], reduction in leaf and primary root growth and photosynthesis in maize [27] and suppressed shoot growth in sorghum [28]. Reduced shoot growth and lower seed yield were reported in the foxtail millet genotype, Maxima, grown under low P in the greenhouse [29]. Thus, the search for genotypes with increased root systems needs to be balanced against potential loss in crop productivity.

In this study, we examined the phenotypic responses of 54 foxtail millet genotypes (*Setaria italica*) to phosphate under phosphate fertilized and unfertilized conditions. Foxtail millet is widely cultivated in the semi-arid regions of Asia (India, China and Japan) as well as in Southern Europe and has the longest history of cultivation among all millets [30]. Recently, it is becoming an increasingly important forage crop in the Americas, Australia and North Africa [31]. Unlike the case with the major cereals, breeding interventions in millets are limited, especially those using biotechnological tools. Foxtail millet is a genetically amenable model crop since it is a diploid and possesses a relatively small genome of ~515 Mb, and, currently, the genome sequence information of two foxtail millet varieties is available [31–33]. However, there are no reports to date exploring the responses of various foxtail millet cultivars to P starvation, except for a regional report [34].

We hypothesised that, given similar culture conditions, with contrasting P levels, those foxtail millet genotypes that fare well under limited P nutrition would be P starvation tolerant and those that do well under high P nutrition would be fertilization responsive. Experimental corroboration of this hypothesis would prove to be of value for future breeding programmes. So, to test this hypothesis, we studied the response of fifty-four foxtail millet genotypes to P sufficiency (P+) and starvation (P-) under both greenhouse and natural field conditions.

## 2 Materials and methods

The field study for each season was carried out on private agricultural lands. We confirm that Mr. Palani, owner of the land, gave permission to conduct the study. We further confirm that no specific permission was required for these selected locations as it is an agricultural land. We also confirm that the field studies did not involve endangered or protected species.

### 2.1 Plant material

A total of 54 genotypes were used in this study, of which 3 genotypes were cultivated locally in southern India, 23 were from other regions in India and the remaining 28 were from 22 other countries, representing a wide geographic coverage. The details of all the fifty-four genotypes are given in S1 Table.

Seeds of 50 of the genotypes were obtained from the International Crops Research Institute for the Semi-Arid Tropics (ICRISAT), Patancheru, Hyderabad, India. Three local genotypes CO5, CO6 and CO7 were procured from the Tamil Nadu Agricultural University (TNAU), Coimbatore, India, and CO5 and CO7 were used as local checks for comparison. The genotype ‘Maxima’ (Acc. No: Bs 3875; Welsh Plant Breeding Station, Genetic Resources Unit, Institute of Grassland and Environmental Research, Aberystwyth University, UK) previously used for genetic characterization on P response [29, 35] was also included in this study. Experiments were conducted under natural field and greenhouse conditions.

### 2.2 Field experimentation

Growth and yield studies were conducted under natural field conditions at Paiyur, Tamil Nadu, India (12°25' N, 78°13' E) located at an elevation of 460 meters above sea level. This location has a history of traditional small millet cultivation for over several hundred years. The selected field is private agricultural land. The owner (Mr Palani) of the land gave permission to conduct the study on this site.

Two separate experiments were conducted under natural field conditions, in two different seasons: the summer of 2015 (April to July) and during the monsoon in 2017 (August to November). The mean temperature during summer 2015 ranged from 32–34°C and during monsoon 2017 it ranged from 28–30°C. The experimental station received an average rainfall of ~105.17 mm from April to July 2015 and ~188.72 mm from August to November 2017. An unfertilized field that was left barren for several seasons was chosen for the study. Prior to the experiment, soil samples were collected from the field, and the available P was found to be 5.5 mg/kg. Under natural field conditions, this is normally considered as Pi-deficient [36].

The crop was raised by sowing the seeds in shallow furrows and plants were grown with a spacing of 30 cm between rows and 10 cm between plants within a row with two replication rows. A block size of 11 x 1 meter was maintained with a spacing of 60 cm between blocks using a randomized complete block design. Each block consisted of 26 genotypes. Either of the two checks, CO5 or CO7, was planted for every three test genotypes. The border rows were planted with check varieties. There were two such blocks per each P regime (P- and P+).

In the P- regime, no P fertilizer was applied and, in the P+ regime, P application was done at double the recommended dose, by applying 100g of diammonium phosphate (DAP) only into each of the P+ plots. To compensate for the extra N supplied through DAP in P+ plots, 40 g of urea was applied in the P- plots such that the amount of N was the same for both Pi treatments. K (25g of muriate of potash which contained 15g of K) was also the same between the Pi treatments. The field was irrigated once a week, and weeding was done every alternate week. The plants were allowed to grow to maturity under normal, recommended agronomic practices. Agro-morphological data were collected on per plant basis at maturity on plant height (PH), tiller number (NT), productive tiller number (NPT), leaf number (NL), leaf length (LL), panicle length (LF), cluster number (NC)/panicle, seeds per cluster (SPC) and total seed yield (TSE)/panicle.

### 2.3 Greenhouse experimentation

To analyse the response of root traits under high P and low P treatments, greenhouse experiments were conducted at the Entomology Research Institute (ERI), Loyola College, Chennai, Tamil Nadu, India. Seeds were sown in 4 L pots (10 seeds/pot) holding 1:1 (v/v) perlite: vermiculite (Astrra Chemicals, Chennai, Tamil Nadu, India) supplied with a basal nutrient solution containing 300 μM of Pi (P+) or 10 μM of Pi (P-) and allowed to germinate. The basal nutrient solution consisted of 2.0 mM Ca(NO_3_)_2_, 0.5 mM MgSO_4_, 0.1 mM KCl, 10 μM H_3_BO_3_, 0.5 μM MnCl_2_, 0.5 μM ZnCl_2_, 0.2 μM CuCl_2_, 0.1 μM Na_2_MoO_4_ and 0.1 mM Fe-EDTA. The Pi concentration was varied by supplying KH_2_PO_4_ while K_2_SO_4_ was used to maintain a constant concentration of potassium in nutrient solutions of differing phosphate concentration [29].

For supplying different Pi regimens in nutrient solutions, different ratios of KH_2_PO_4_ and K_2_SO_4_ were used to maintain a constant concentration of potassium in nutrient solutions. Three replicates were maintained for each of the Pi treatments. The pots were maintained in a greenhouse at 26ºC, 16 h light with 85% relative humidity under well-lit and aerated conditions. The nutrient solutions were supplied once in three days.

The shoot dry weight (SDW) and root dry weight (RDW) (whole root system) of 15-day old seedlings for each treatment were observed and combined into a single trait, biomass, to avoid reduced variance due to fractionation. Shoot length (SL), root length (RL) (primary root), root hair density (RHD) and root hair length (RHL) were determined at 28^th^ day. For the analysis of RHD and RHL, the seedlings were carefully removed from perlite, washed well with distilled water and blotted dry. For measurements, five roots tip samples were drawn from the young fully grown radially equidistant secondary roots. Root hair measurements were performed according to Slabaugh et al., [37] with some modifications. The part about 5 cm from the primary root cap was photographed using a Canon Coolpix digital camera under a Leica Stereo Microscope (Wetzlar, Germany) and measured using ImageJ image processing and analysis software v1.80_112 [38].

### 2.4 Analysis of phosphate content

In the field experiment, based on phenotypic performance, five highest (low performers) and five lowest (high performers) ranking genotypes were picked from the extremes of the total phenotypic rank distribution. The selection of genotypes at both extremes was done independently on both P- and P+ treatments following the same procedure. Similarly, genotypes with extreme values were picked based on greenhouse performance under both P- and P+ treatments independently. Shoot and root tissues of the 4-week old genotypes grown under greenhouse and field conditions were used for the analysis of total and inorganic P (Pi) contents. The total P and Pi contents were analysed using the protocol reported previously by Chiou et al. [39].

### 2.5 Data analyses

Analysis of variance was conducted on field data for two seasons using the restricted maximum likelihood (REML) approach implemented in the lme4 package in the R statistical environment. Genotypes and season were treated as random factors to estimate unbiased predictors from the model. Traits having non-significant genotypic effects were dropped, and the predicted genotype effects of different traits were used for further analyses. To identify the 10% extreme genotypes for overall phenotypic performance, trait predictors were ranked in descending order, individually for all traits, so that the highest value gets rank one. The cumulative ranking (rank sum) of each genotype was computed by adding the ranks of that genotype for all traits. For instance, under P+, the genotype ISe 1387 was ranked 1^st^ for PH, NL, LF, NC and TSE, 2^nd^ for LL, 3^rd^ for SPC and 37^th^ for NPT making its rank sum 47. The rank sums were similarly worked out for the rest of the genotypes. A similar approach was implemented for the greenhouse data.

From the rank sums, five extreme genotypes, having low- and high-rank sums, were picked under each category of P- and P+ treatments. Four classes of extreme genotypes were selected: lowest rank sum (high performers) under P+, highest rank sum (low performers) under P+, high and low performers under P-.

### 2.6 Genotype comparison for P response under field and greenhouse conditions

Field and greenhouse data were used to identify genotypic similarity in P response both under P+ and P- conditions. Before clustering, the data were centred by subtracting the trait means and scaled by dividing by the respective standard deviation. A popular unsupervised centroid-based clustering known as k-means clustering [40] was executed in R using the *kmeans* function. The clusters were graphically represented using the package, gplots.

### 2.7 Cluster analysis based on plasticity

Response to P availability in plants was assumed to affect all the phenotypic traits observed. Therefore, the genotypic P response among different traits (plasticity) was worked out as the percentage difference between both P conditions over the mean P response. Hierarchical clustering was done on the plasticity matrix using correlation as the distance measure and by performing multiscale bootstrap resampling [41]. The *p*-values computed for bootstrap events for each cluster were used for identifying significant clusters. There are two *p*-values computed: the bootstrap probability, BP, and the approximately unbiased *p*-value, AU, a better measure of approximation [41, 42]. The computations were performed with the pvclust package in R, version 2.2–0 [43] using correlations and average agglomeration with 3000 bootstrap resamplings. All the analyses were done using RStudio 1.1.463 running R version 3.5.1. The R script used for creating figures are given in the supplementary file (S4 Table).

## 3 Results

### 3.1 Foxtail millet shows significant genotypic variation for all the traits under field conditions

Analysis of variance revealed that significant genotypic variation existed for all the traits under both P- and P+ conditions (Table 1). The season and genotype x season component also showed non-significant variance. The heritability and error variance showed profound influence of genotypic variance on the total variance for all the traits in the population. These estimates are indicative of the general trends in the influence of different sources of variation on the heritable component of the trait [44].

**Table 1:** Testing for the significance of variance component effects by restricted maximum likelihood (REML) method.

Traits	Variance			AIC	Heritability
	Genotype	Season	Genotype x Season		
H-PH	269.5*	7.50	4.05	2701.53	0.95
H-NPT	5.68*	0.11	0.54	1638.07	0.91
H-NL	1.21*	0.05	0.00	1251.44	0.87
H-LL	37.8*	0.34	0.64	2085.77	0.95
H-LF	16.23*	0.48	1.05	1702.24	0.97
H-NC	270.73*	4.21	5.98	2446.55	0.97
H-SPC	379.2*	4.52	34.4	2310.76	0.95
H-TSE	41.12*	0.32	0.18	880.99	0.99
L-PH	511.32*	5.46	2.05	2307.75	0.98
L-NPT	6.54*	0.32	0.02	1503.11	0.92
L-NL	1.99*	0.03	0.00	1120.4	0.93
L-LL	52.24*	0.26	0.04	1918.04	0.95
L-LF	15.6*	0.34	0.47	1531.03	0.96
L-NC	344.5*	14.04	9.74	2104.48	0.97
L-SPC	285.97*	1.62	6.61	1905.93	0.98
L-TSE	45.76*	0.42	0.21	656.35	0.99

*Significant at p<0.05 by chi-square test. AIC, Akaike information criterion. Trait names are prefixed H- for P+ response and L- for low P response. PH, plant height in cm; NT, number of tillers; NPT, number of productive tillers; NL, number of leaves; LL, length of leaf in cm; LF, length of panicle in cm; NC, number of clusters/panicle; SPC, seeds per cluster; TSE, total seed yield in g.

### 3.2 Cumulative ranking of foxtail millet genotypes for all the significant traits under field conditions

**Fig 1:**
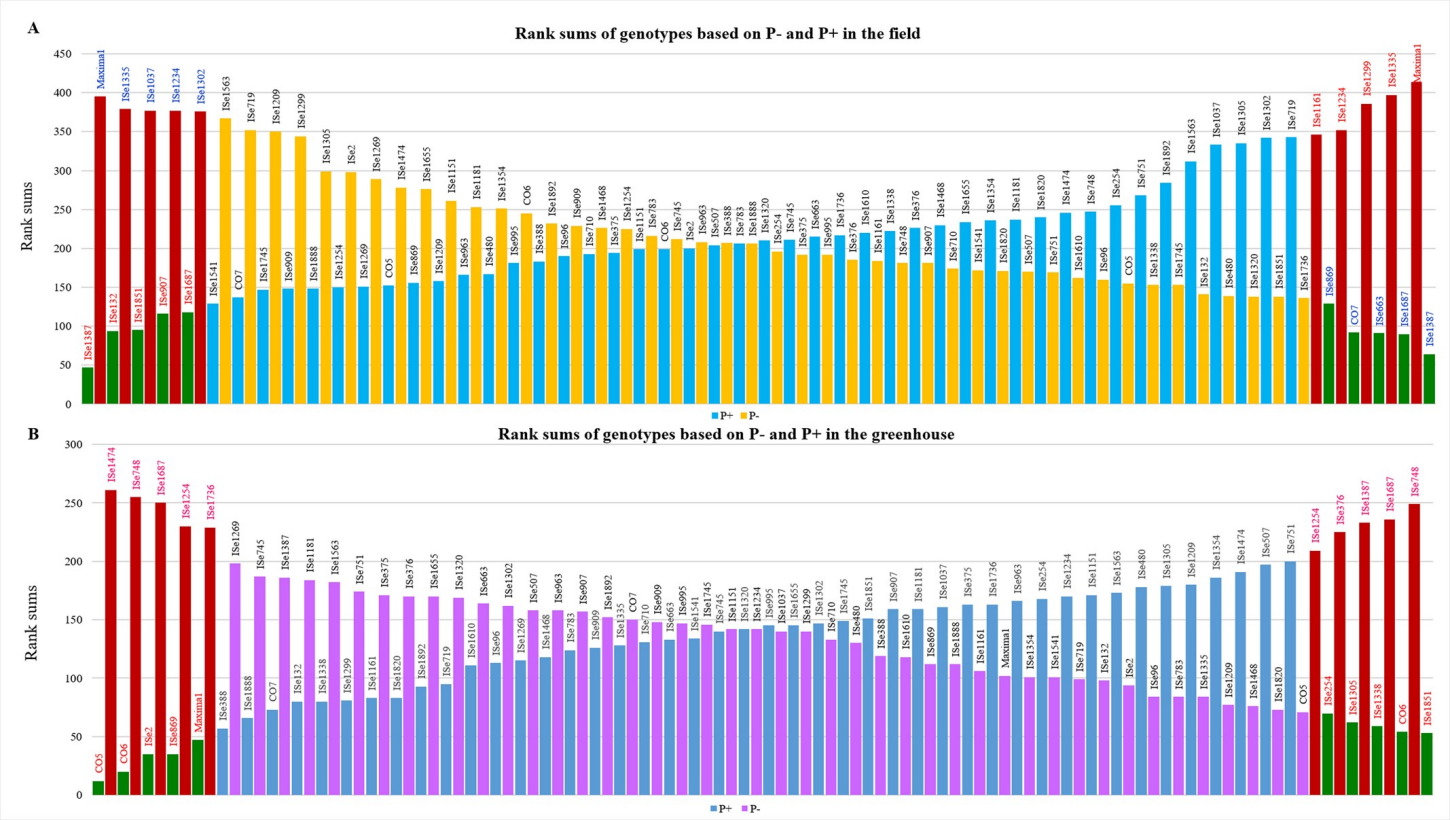
Rank sums of genotypes based on (A) field and (B) greenhouse evaluation. The 10 extreme genotypes are selected under each P regime, P+ and P-. P+ ranks are ordered from poor (having high-rank sum) to good (having low-rank sum). P ranks are ordered from good (low-rank sum) to poor (high-rank sum). Five genotypes with the lowest rank sum in each group are selected as best genotypes (green), and five genotypes each with highest rank sum are selected as poor genotypes (red).

Based on the cumulative ranking, genotypes ISe 1387 and ISe 1687 were in the top 5 in both Pi fertilized and unfertilized field plots (Fig 1A). Other genotypes that performed well under both P- and P+ conditions were CO7 (4^th^, 7^th^), ISe 132 (10^th^, 2^nd^), ISe 1851 (7^th^, 3^rd^) and ISe 869 (5^th^, 14^th^). Other top genotypes were ISe 663 (3^rd^ under P-) and ISe 132 and ISe 907 (2^nd^ and 4^th^ under P+, respectively). Maxima followed by ISe 1335 and ISe 1234 showed poor performance in the field under both P- and P+ conditions. The other genotypes that were poor under P- are ISe 1037 and ISe 1302.

### 3.3 Foxtail millet genotypes show differential responses to Pi under natural field and greenhouse conditions

The mean values of the responses of each trait for all the genotypes analysed in the field experiments are given in Table 2 and S2 Table. When the good and poor performing genotypes were compared, based on the rank sums for the P+ plot, statistically significant differences were seen for all the traits. Even in the case of P- conditions, conspicuous differences were recorded for all the traits. However, when the selection was done for extreme five genotypes in both the directions in both the treatments, differences in phenotypic performance were found to be reduced.

**Table 2:** Mean values of the five high and low responding genotypes of foxtail millet in growth assays under unfertilized (P-) and P fertilized (P+) natural field conditions.

Genotypes	PH	NPT	NL	LL	LF	NC	SPC	TSE
**Good responders under unfertilized plot (P-)**								
ISe 1387*	146.33±17.41^a^	4.5±1.76^a^	10.5±1.05^a^	47.71±3.28^a^	23.93±2.83^a^	142.33±15.86^a^	49.87±8.96^a^	25.15±1.31^a^
ISe 1687*	133±10.77^b^	8.33±1.75^b^	10±1.26^b^	39.21±9.82^b^	17.75±2.31^b^	74.67±8.64^b^	60.33±6.8^b^	14.79±0.9^b^
ISe 663*	131.17±7.83^b^	13.33±1.37^c^	10.67±0.82^b^	32.56±3.08^c^	20.45±1.55^c^	79±8.41^c^	28.33±3.72^c^	17.93±1.01^c^
CO7*	132.86±4.6^b^	10.97±1.47^d^	10±0.63^c^	35.45±1.65^d^	20.96±0.92^c^	85±2.34^d^	30.33±2.94^c^	14.13±1.09^b^
ISe 869*	140.83±14.96^c^	8.67±3.44^e^	9.17±1.6^d^	29.21±6.46^e^	18.46±3.78^d^	73.67±13.69^b^	41.5±5.01^d^	12.66±0.48^d^
**Poor responders under unfertilized plot (P-)**								
**Maxima**^**#**^	72.83±10.4^a^	4.33±1.21^a^	6.33±1.51^a^	17.3±3.75^a^	8.83±5.4^a^	28.33±12.24^a^	20.04±1.89^a^	1.15±0.38^a^
**ISe 1335**^**#**^	66.83±16.49^b^	5.0±1.79^b^	5.17 ± 1.6^b^	11.51±4.05^b^	5.96±2.4^b^	33.83±3.66^b^	32.5±5.05^b^	2.28±0.61^a^
**ISe 1037**^**#**^	68.33±17.11^b^	4.83±2.14^b^	7.33±1.75^c^	19.23±6.68^c^	8.9±1.78^c^	45.33±5.2^c^	10.83±2.32^c^	4.28±0.79^b^
**ISe 1234**^**#**^	72.67±17.49^a^	5.17±1.94^c^	6.33±1.63^a^	18.63±7.21^d^	8.89±3.73^c^	48.33±5.92^c^	11.33±1.21^c^	3.84±0.62^b^
**ISe 1302**^**#**^	80±15.57^c^	5.17±1.6^c^	6.5±0.84^a^	18.15±2.58^d^	10.01±2.12^d^	40.5±3.21^d^	14.67±3.14^c^	3.04±0.18^b^
**Good responders under P fertilized plot (P+)**								
ISe 1387*	173.17±16.67^a^	6.67±1.37^a^	11.67±1.75^a^	48.26±8.14^a^	27.63±3.2^a^	152.67±16.28^a^	90±11.76^a^	30.52±1.15^a^
ISe 132*	142.83±8.04^b^	10.67±5.5^b^	8.17±1.47^b^	35.86±3.74^b^	23.25±1.78^b^	82±6.16^b^	29.5±5.96^b^	28.48±1.43^b^
ISe 1851*	151.5±15.64^c^	7±2.37^c^	9.5±0.55^c^	33.89±3.87^c^	21.46±2.68^c^	69.67±7.99^c^	43.83±4.4^c^	22.39±1.08^c^
ISe 907*	131±10.04^d^	10.67±3.33^b^	8.33 ± 0.52^b^	39.87±13.54^d^	18.77±3.21^d^	80.5±1.52^b^	40.13±11.14^c^	15.91±0.47^d^
ISe 1687*	131.33 ± 5.68^d^	8.83±3.19^d^	9 ± 1.26^d^	33.1±3.79^c^	19.61±2.61^d^	68.17±9.06^d^	74±6.23^d^	15.8±0.76^d^
**Poor responders under P fertilized plot (P+)**								
**Maxima**^**#**^	87.17±16.8^a^	4±1.67^a^	5±1.41^a^	16.48±6.01^a^	9.05±3.38^a^	34.33±8.85^a^	25.89±2.4^a^	0.83±0.34^a^
**ISe 1335**^**#**^	84.33±12.55^a^	5±1.67^b^	5.17±2.32^a^	12.52±5.68^b^	6.41±1.14^b^	29.67±9.73^b^	32±4.6^b^	2.94±0.4^b^
**ISe 1299**^**#**^	83.17±8.4^b^	7.5±0.84^c^	6.83±0.41^b^	22.54±3.04^c^	10.03±3.17^c^	47.83±7.39^c^	12.83±1.94^c^	2.97±0.41^b^
**ISe 1234**^**#**^	93.67±5.39^c^	4.67±2.5^d^	6.17 ±1.33^c^	22.86±4.22^c^	9.03±3.43^a^	41.5±8.17^d^	22.33±3.27^d^	28.26±0.87^c^
**ISe 1161**^**#**^	107.67±23.31^d^	9.17±0.75^e^	6.67±2.25^b^	21.34±7.39^c^	13.65±4.33^d^	53.33±15.81^e^	29.5±6.66^a^	5.98±0.13^d^

Values are Mean ± SD

*, high ranking genotypes

#, low ranking genotypes. The genotypes showing variation between total and productive tillers are in bold. In each column, values followed by different letters are significantly different (*p<0*.*05*) based on students t-test. PH, plant height in cm; NT, number of tillers; NPT, number of productive tillers; NL number of leaves; LL, length of leaf in cm; LF, length of panicle in cm; NC, number of clusters/panicle; SPC, seeds per cluster; TSE, total seed yield in g.

In total five traits were analyzed in greenhouse grown plants (Fig 1B). ISe 1387, which was the highest-ranking genotype under field conditions in both Pi regimes, ranked low when grown under greenhouse conditions. Maxima, which showed a poor field performance under both fertilized and unfertilized conditions, was the fourth best performer under P+ treatments, and mid-ranking under P- in the greenhouse. Similarly, CO 6, which performed very well under both P+ and P- in the greenhouse experiments, indicated a moderate response under field conditions (37^th^ under P- conditions and 24^th^ under P+ conditions). ISe 1851 performed well under both field as well as greenhouse conditions and was the most consistent high-responding genotype in the study. Other high performers were CO7 and ISe 869. Further, the genotype CO5 which was placed at the 13^th^ position in both P- and P+ soil, was placed at the first position under P+ and 6^th^ position under P- situations under greenhouse evaluation.

Greenhouse-grown plants also showed a high degree of variation under differing levels of Pi (S3 Table). Genotype ISe 1851 had the lowest rank sum (high performer) under P-, followed by CO6, ISe 1338, ISe 1305 and ISe 1254 (Fig 1B). Genotypes ISe 1474 and ISe 748 had the highest (low performer) rank sum under P-. Under high phosphate, the cumulative rank order was very different. The genotype CO6 was 2nd under P+. Genotype ISe 748 performed poorly under P+ as well as P- (Fig 1B). Collectively, the high responding genotypes under P- conditions had significantly higher biomass, SL, RL, RHD and RHL than low performers (Table 3). The low performer, ISe 1687, registered significantly higher RHD and RHL than other low performers under P- condition, whereas RHL and RHD are comparable to those of high performers.

**Table 3:** Mean values for the five high and low responding genotypes of foxtail millet in growth assays under low (P-) and high (P+) in greenhouse conditions.

Genotypes	BIO	SL	RL	RHD	RHL
Good respo*nders under P+					
CO-5*	8.00±1.00^a^	21.73±7.98^a^	13.13±0.87^a^	41.67±4.04^a^	3.16±0.01^a^
CO-6*	10.47±0.15^b^	15.87±5.24^b^	19.60±0.82^b^	21.67±3.06^b^	3.26±0.00^a^
ISe 2*	7.10±1.00^c^	17.30±0.61^c^	15.00±1.00^c^	13.67±1.53^c^	3.04±0.04^a^
ISe 869*	6.70±0.62^c^	18.07±5.97^c^	8.80±1.25^d^	23.33±2.52^d^	3.56±0.07^b^
Maxima*	15.30±1.00^d^	10.20±2.35^d^	10.60±3.08^e^	24.67±3.51^d^	2.57±0.04^c^
**Poor responders under P+**					
**ISe 748#**	2.13±0.60^a^	2.60±0.92^a^	5.17±0.40^a^	0.00±0.00	0.00±0.00
**ISe 1687#**	2.80±2.27^b^	5.17±1.67^b^	5.40±0.26^ab^	1.00±1.73^a^	0.49±0.00^a^
**ISe 1387#**	3.00±0.56^b^	4.53±0.95^b^	6.70±0.62^c^	0.00±0.00	0.00±0.00
**ISe 376#**	3.50±0.30^c^	8.43±2.40^c^	5.93±1.55^b^	0.00±0.00	0.00±0.00
**ISe 1254#**	6.10±1.15^d^	2.93±0.51^a^	4.87±0.67^ab^	1.67±2.89^a^	0.40±0.00^a^
**Good responders under P-**					
ISe 1851*	7.03±3.67^a^	9.13±2.81^a^	12.43±3.77^a^	51.33±3.21^a^	4.85±0.10^a^
CO-6*	8.00±0.46^c^	14.40±3.27^b^	13.70±1.62^b^	45.33±3.79^b^	3.70±0.11^b^
ISe 1338*	6.66±0.32^a^	15.90±2.25^c^	16.10±2.21^c^	49.33±1.53^a^	3.75±0.12^b^
ISe 1305*	6.07±2.15^b^	13.00±0.50^d^	9.50±4.74^d^	50.67±4.51^a^	6.24±0.05^c^
ISe 254*	5.87±0.81^b^	10.43±4.56^e^	9.13±1.21^d^	44.00±3.61^b^	8.01±0.10^d^
**Poor responders under P-**					
**ISe 1474#**	2.07±0.59^a^	4.50±2.18^b^	2.47±0.45^a^	1.20±0.00^a^	1.06±0.18^a^
**ISe 748#**	2.90±0.36^b^	5.90±1.91^b^	1.67±0.76^b^	0.00±0.00	0.00±0.00
**ISe 1687#**	2.40±1.01^a^	5.17±2.02^b^	3.50±1.37^c^	31.00±3.00^b^	2.37±0.03^b^
**ISe 1254#**	4.43±0.78^c^	6.67±0.38^c^	4.73±0.25^d^	0.00±0.00	0.00±0.00
**ISe 1736#**	3.90±0.96^d^	5.40±1.89^b^	7.10±1.21^e^	0.00±0.00	0.00±0.00

Values are Mean ± SD

* = High ranking genotypes

# = Poor ranking genotypes. Within each column, values followed by different letters are significantly different (*p<0*.*05*) based on students t-test. BIO, seedling biomass in mg; SL, shoot length in cm; RL, root length in cm; RHD, root hair density per 10μm length, RHL, root hair length in μm.

### 3.4 Root hair density and root hair length are associated with better PUE

**Fig 2:**
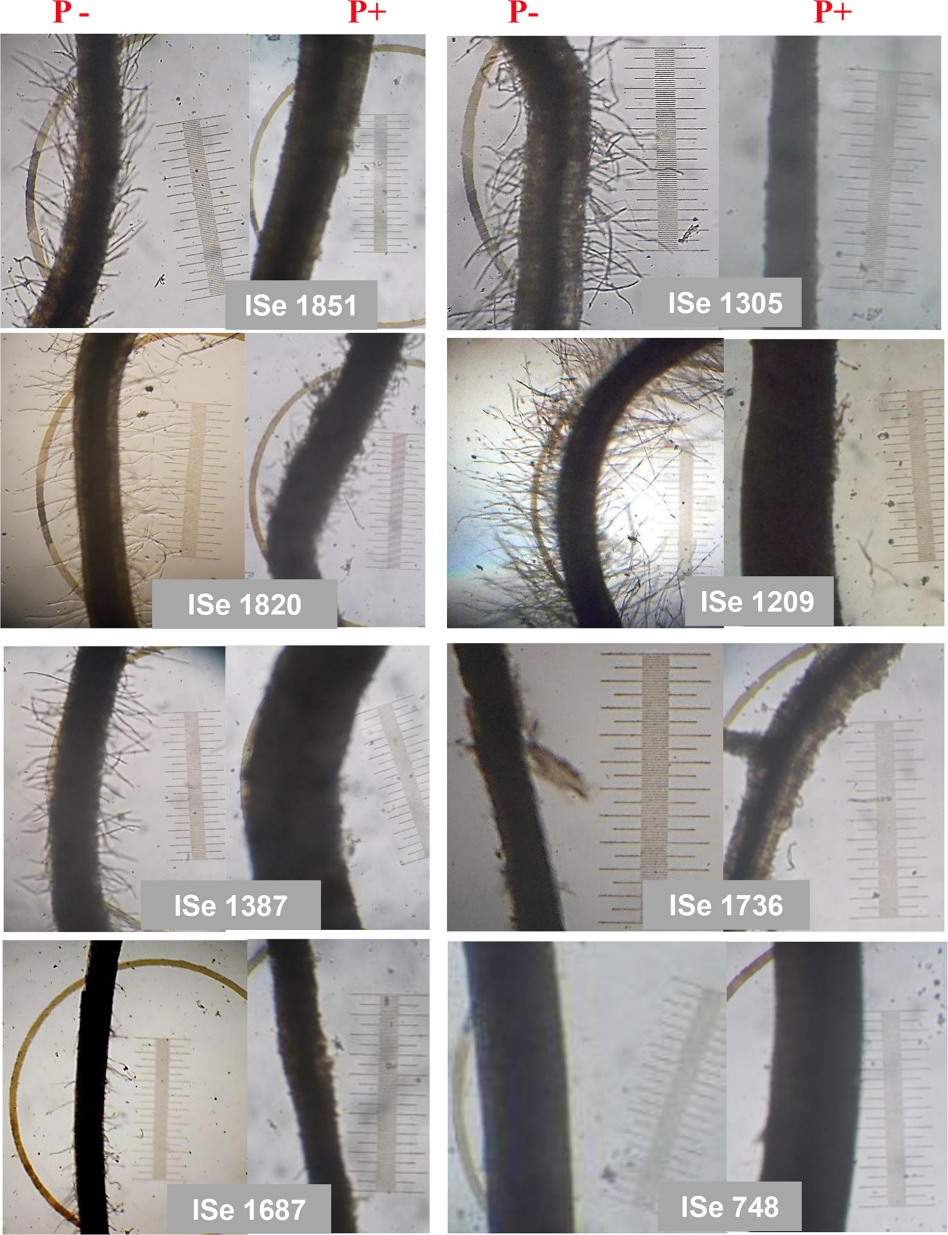
Root hair images of selected genotypes of foxtail millet showing a response to P- and P+ in the greenhouse. The image was taken after 15 days of growth under P- and P+ in the greenhouse. The genotypes ISe 1851 and ISe 1305 are on the top in the cumulative ranking and high responding genotypes for root hair formation under P- condition. The genotypes ISe 748, ISe 1687 and ISe 1736 are low performers under P- in greenhouse. The genotype ISe 1387 is a low performer under P+ in greenhouse. The genotype ISe 1820 is an intermediate responder in both P- and P+.

Under P+ conditions, the five low performers were comparable to the five low performers under P- conditions for all parameters. Genotypes ISe 748, ISe 1254 and ISe 1687 were common to both treatments as low performers. ISe 748, ISe 1254 and ISe 1736, grown under P+ treatment as well as under P- condition, lacked root hairs (Table 3 and Fig 2**).** In contrast, the root hairs in the other genotypes (ISe 1851, ISe 1305, ISe 1209 and ISe 1387) shown in Fig 2 were sparse or indiscernible under P+ conditions but were increased in length and number in P-conditions. The high responding genotypes under P+ were different from the high responding genotypes under P- with the exception of the local genotype CO6, which was ranked 2^nd^ under both P- and P+ (Fig 1B). Under P- conditions, CO6 attained 90% of the SL and 76% of biomass seen under P+. For the root traits under P-, CO6 attained 69% of the RL, 209% of the RHD and 113% of the RHL attained under P+ conditions.

All the high responding foxtail millet genotypes (ISe 1851, CO6, ISe 1338, ISe 1305 and ISe 254) produced abundant and long root hairs under P- in the greenhouse experiment. RHL and RHD were associated with higher Pi content under P- conditions and Pi content is a good indicator to gain better information on plant response under low P conditions. We also assessed Pi uptake in selective genotypes which confirmed that RHL and RHD were associated with Pi uptake under P- conditions. Fig 2 shows the extent of variability of the root hairs in the high and low responders under P- conditions in greenhouse experiments.

### 3.5 P content of root and shoot tissues of high-responding genotypes were higher than those of low performers under field and greenhouse conditions

**Fig 3:**
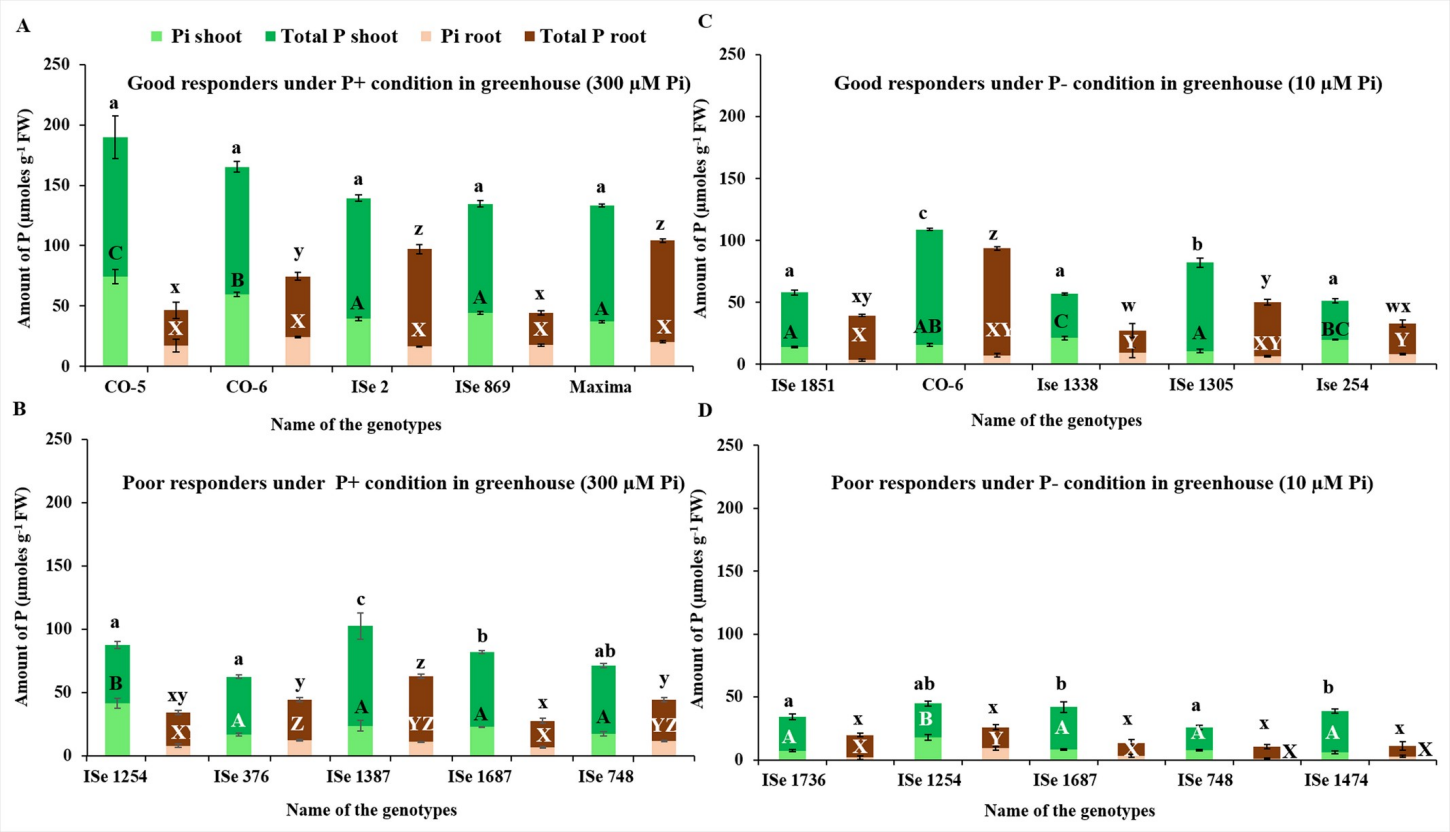
Inorganic (green) and total P assay (brown) for each of the five high performers and low performers of foxtail millet genotypes under P- (10 μM Pi) and P+ (300 μM Pi) conditions in greenhouse conditions. A, high performers under P+; B, low performers under P+; C, high performers under P-; D, low performers under P-. The letters over the bars indicate statistically significant differences if the letters are different as p<0.05. The vertical lines indicate error bars.

**Fig 4:**
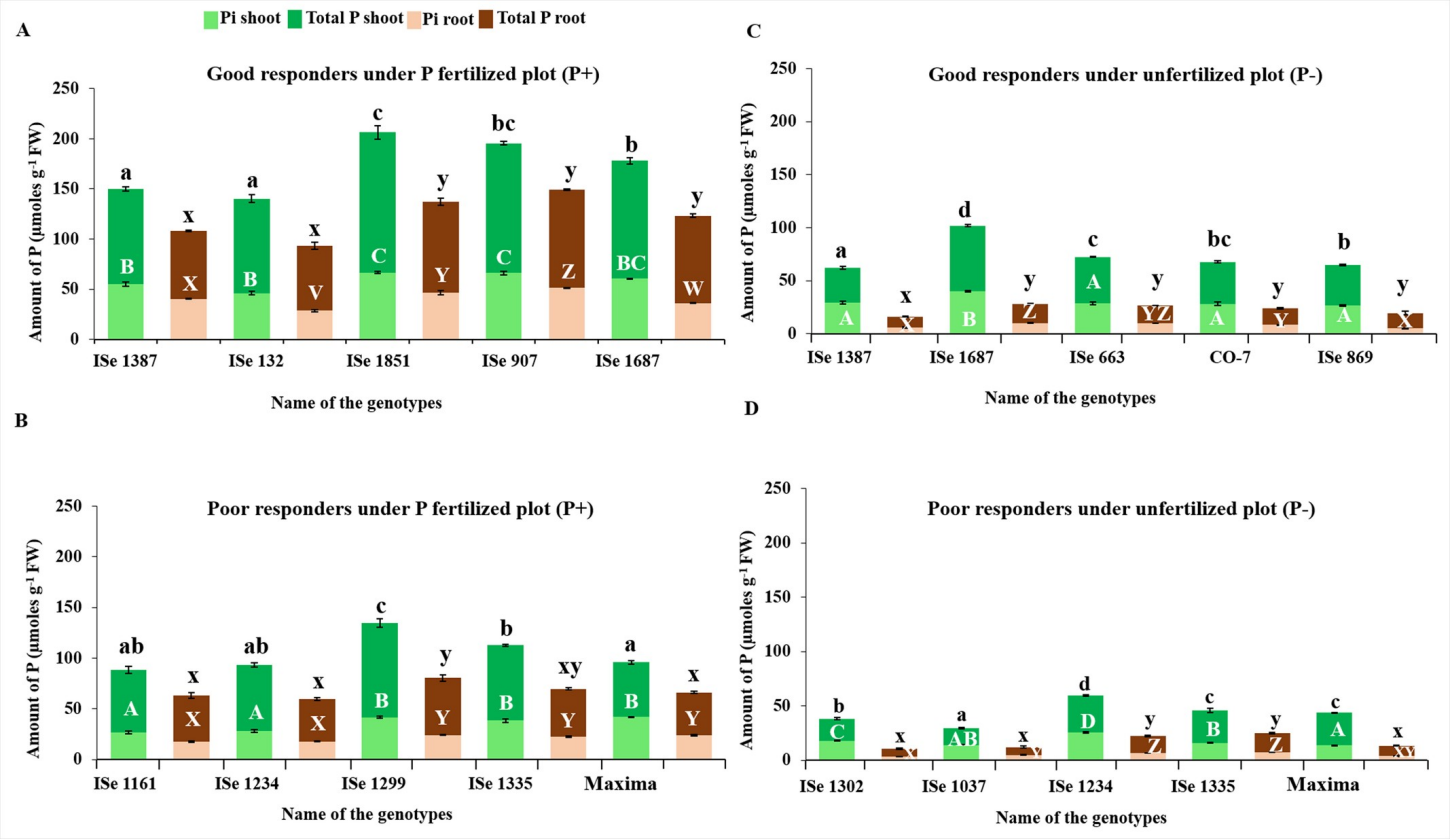
Inorganic (green) and total P assay (brown) for the five each of high performers and low performers of foxtail millet genotypes under unfertilized (P-) and P fertilized (P+) conditions in the natural field. A, high performers under P+; B, low performers under P+; C, high performers under P-; D, low performers under P-. The letters over the bars indicate statistically significant difference if the letters are different as p<0.05. The vertical lines indicate error bars.

The total P and Pi contents were assayed in the leaf and root tissues of each of the five high and low responding genotypes under P- and P+ conditions, grown in the greenhouse (Fig 3) as well as in the field (Fig 4). As expected, in both high and low performers, more plant Pi is present under P+ conditions (Fig 3A and 3B) than under P- conditions (Fig 3C and 3D). Good responders under the P+ condition in the glasshouse generally had much higher (1.5-3- fold higher) total leaf P and leaf Pi contents than low performers. Good responders on P- maintained similar levels of tissue P as poor responders on P+ suggesting higher PAE (compare Fig 3B and 3C). Under P-, the high responding genotypes maintained higher levels of both root and shoot P compared to poor responders (Fig 3C and 3D).

Root total P and Pi was also higher although there was more variation and some overlap with some of the poor responders such as ISe 748 and ISe 1387 (Fig 3A and 3B). The low-responding genotype ISe 748 had the same level of Pi in root tissues as some of the high-responding genotypes (CO5 and ISe 869) but much less in the shoot tissues. This may suggest that ISe 748 had a low ability in transporting Pi from root to shoot tissues (compare Fig 3A and 3B).

Under field conditions, the best performers had higher (>50%) total and Pi content than the poor performers (Fig 4A and 4B). The levels of shoot total P and Pi were similar between the good responders under glasshouse and field conditions, but root total P and Pi was higher in the best performers under field conditions than in glasshouse (Fig 3A and 4A). The good responders grown under P- condition in the field-maintained levels of total P and Pi in shoots similar to those poor responders grown under P+ condition (compare Fig 4C and 4B). The good responders on P- maintained levels of leaf Pi similar to poor responders on P+ but root total P and Pi was much lower, again pointing to the role of effective transport from root to shoot (Fig 4B and 4C). The high responding genotype ISe 1687, for example, had more than 100 μmol/g total P, significantly greater than found in other high performers and all low performers under P- treatment.

Interrelations between P content among the extreme genotypes showed that shoot and root Pi contents were significantly correlated to total P in the respective tissues both under field and greenhouse screening (Table 4). Under greenhouse situations, Pi content of shoot tissues was also related to root Pi content but not to total root P content. Overall, under field situations, both the root Pi and shoot Pi were correlated as well as to the total P content in both the plant tissues. Conspicuously, there was good agreement between P content of the same genotypes grown under field and greenhouse conditions in both the root and shoot tissues. Analysis of the correlations between tissue-specific P content assayed under field and greenhouse, within different categories of genotypes falling within the four response classes indicated discernible deviation from the overall pattern. There was no apparent difference between the shoot Pi of the good and poor responders under P- condition in field and greenhouse conditions. However, under P+ conditions, there was a significant but negative correlation. This negative correlation between field and greenhouse-based assays was reflected in total shoot P as well as root Pi, under P+ regime. In poor performers, however, total root and shoot P showed a significant positive correlation between field and greenhouse data.

**Table 4:** Interrelations between P content in shoot and roots among the extreme genotypes showing P response under greenhouse (upper diagonal) and field (lower diagonal) conditions. The diagonal values (in bold) are correlations between greenhouse and field parameters. Values shown against genotype response groups are the correlations between field and greenhouse parameters under each category.

P response under field	P response under greenhouse				
	* *	*Pi shoot*	*Total P shoot*	*Pi root*	*Total P root*
	Pi shoot	0.705*	0.761*	0.820*	0.294
	Total P shoot	0.949*	0.842*	0.790*	0.730*
	Pi root	0.940*	0.953*	0.808*	0.526*
	Total P root	0.919*	0.971*	0.978*	0.501*
	Good P-	-0.157	0.747*	0.407	0.418
	Poor P-	-0.250	0.215	-0.190	-0.692*
	Good P+	-0.668*	-0.746*	-0.878*	0.237
	Poor P+	-0.507*	0.870*	0.066	0.698*

*significant as p<0.05

### 3.6 Variations of inflorescence in response to P+

**Fig 5:**
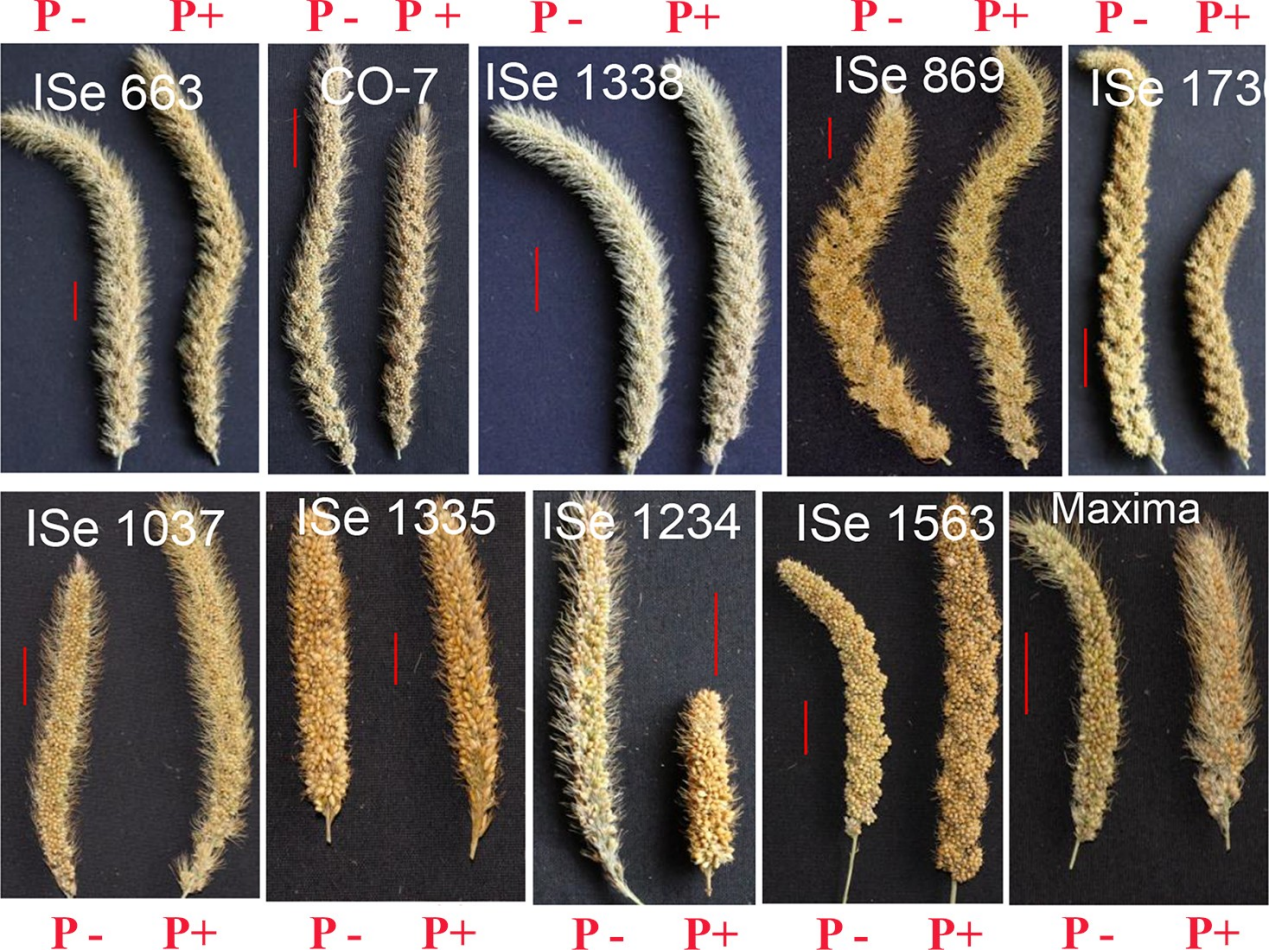
Flowers with mature seeds of selected genotypes of foxtail millet grown in a natural field, Pi fertilized (P+) and unfertilized (P-) soil conditions (P-, low phosphate; P+, high phosphate). The genotype ISe 663 is low P tolerant, the genotypes ISe 1234 and ISe 1563 are high P tolerant, and the genotypes CO-7, ISe 1335, ISe 1338, ISe 869, ISe 1736, ISe 1037 and Maxima are intermediate response under both P+ and P- conditions based on the analysis of genotypic plasticity for seed yield.

The inflorescence images depicting the variation in the responses of some of the genotypes to P fertilization are given in Fig 5. Generally, it is expected that the plants grown under low P should produce small inflorescence when compared to P+ conditions. As can be seen in Fig 5, some genotypes (e.g. ISe 663, ISe 869, ISe 1037, ISe 1563) indeed respond with an increased size of the inflorescence. However, some genotypes (e.g. ISe 1234, ISe 1736, CO-7 and Maxima) respond to high P with reduced size of inflorescence. Since we used double the recommended dose of P in P+ fields and greenhouse experiments, the optimum P varies from genotype to genotype. Higher than the optimum P level may be responsible for the decreased inflorescence size. This implies that there is a need to optimise the use of P for each genotype to get maximum yield.

### 3.7 Genotypic plasticity for seed yield identifies low P tolerant genotypes based on P response

**Fig 6:**
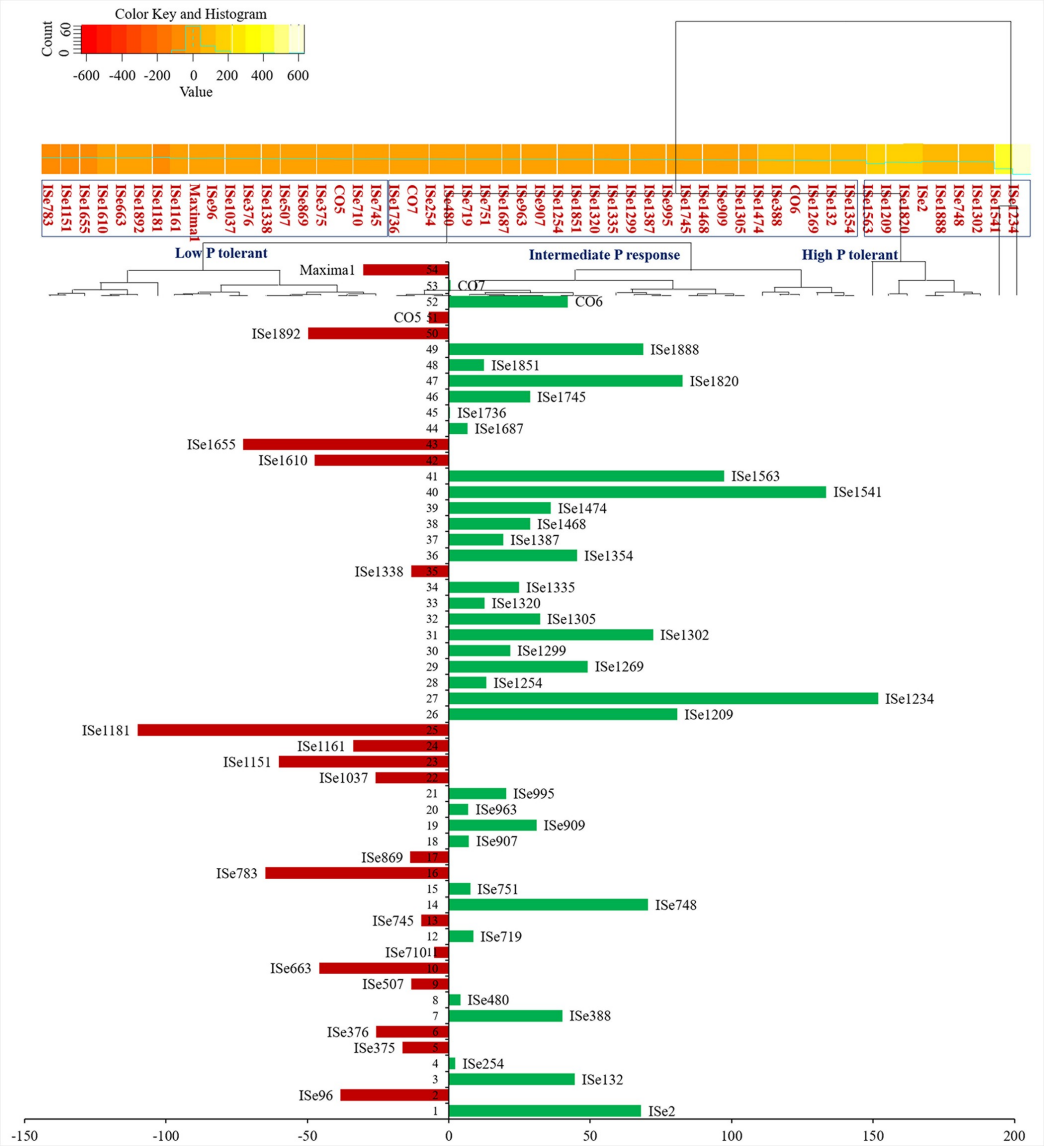
Differential response of genotypes for total seed yield. The genotypes on the left side of the figure (Scale 0 to -150 and red colour bars) showed tolerance to low P. Genotypes with long bars on the right side of the figure (Scale 0 to 200 and green colour bars) are P+ responders, those closer to the axis are more plastic to P level variations and show stable performance under both P+ and P- conditions.

As a crop, we are interested in the response of foxtail millet genotypes to P in terms of seed yield, more than in terms of the other traits examined so far: root, shoot and inflorescence. Genotypic plasticity, the ability of plants to withstand extreme variations in P availability, could identify three sets of genotypes such as low P tolerant, high P tolerant (P+ responders) and intermediates under both P+ and P- conditions. The genotypes ISe 1181, ISe 1655, ISe 783, ISe 1892 were low P tolerant based on seed yield (on the left side of Fig 6). Genotypes ISe 1234, ISe 1541, ISe 1563, ISe 1820, and ISe 1888 were P+ responders and hence were identified as high P tolerant types (on the right side of Fig 6).

Although plasticity over the mean performance was low (19.3%), the genotype ISe 1387 showed a particularly strong response to P application in both greenhouse and field conditions, in terms of absolute phenotypic performance (Figs 4 and 5). However, this genotype was high P tolerant, not low P tolerant based on plasticity analysis of total seed yield (Fig 6).

### 3.8 Genotype clusters based on P level response in field and greenhouse experiments

**Fig 7:**
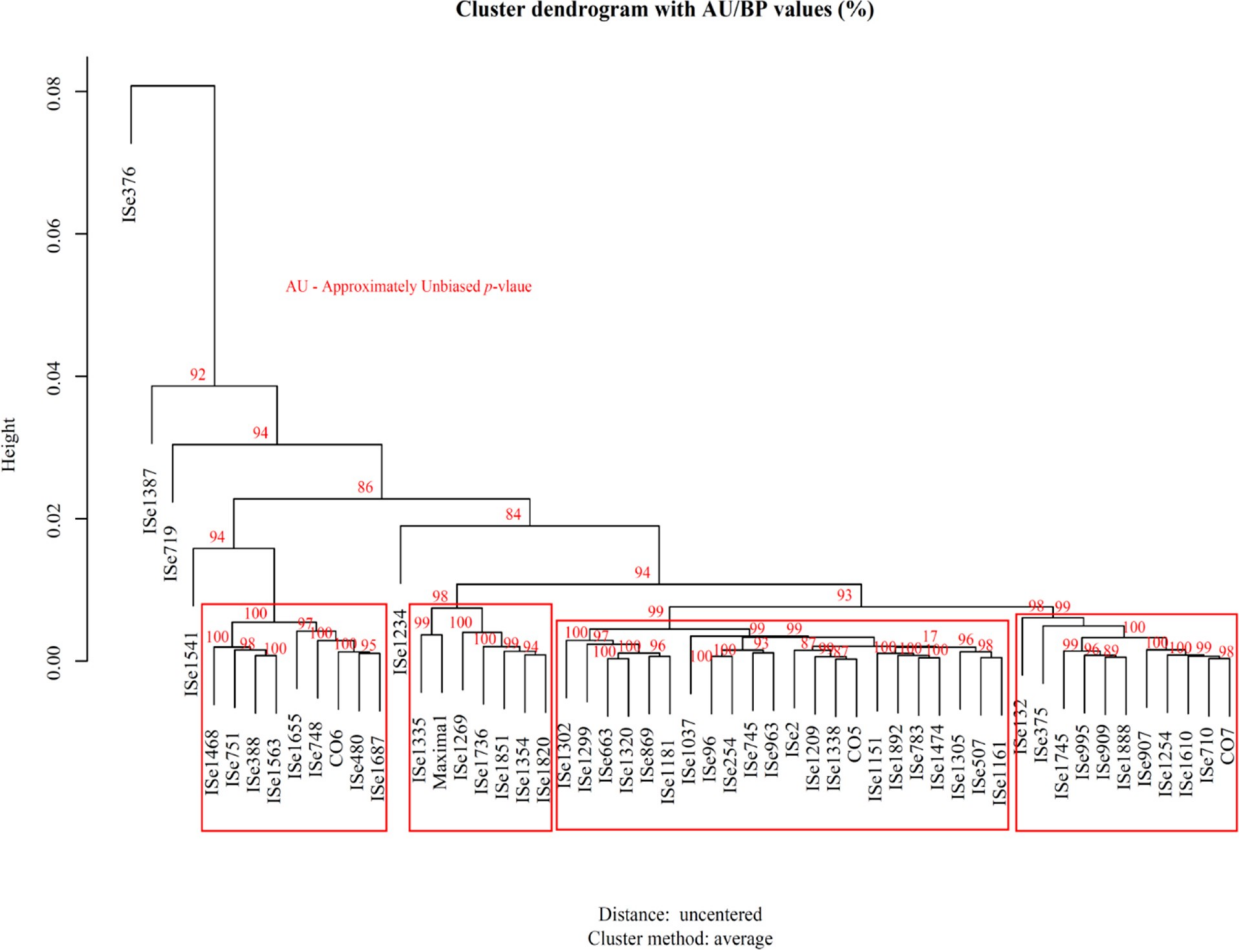
Hierarchical cluster showing the ratio of approximately unbiased probability (AU) for the P response genotypes and bootstrap probability expressed in percentage. When the ratio is greater than 95%, it indicates that clusters created by the variables of P response and node is strongly supported by data. Red boxes demarcate the clustering of the genotypes.

The standardised phenotypic data-based analysis of 54 foxtail millet genotypes, in both greenhouse and field conditions under P- and P+, revealed two to four clusters of genotypes by *k*-means clustering. The cluster pattern was displayed as a heatmap in S1 Fig, S2 Fig, S3 Fig and S4 Fig, where yellow equates to a high value and red to a low one. Comparing performance in the field, some of these varieties showed that variabilities in agronomical traits patterned the grouping of foxtail millet genotypes in response to Pi conditions. There were four clusters of genotypes under P- field conditions (S1 Fig). The genotypes ISe 1181, ISe 375 and ISe 376 were low P responders for total seed yield, and these three genotypes were found grouped together into one cluster (S1 Fig). However, these three genotypes were grouped in separate clusters based on the bootstrap probability (BP) and the approximately unbiased (AU) *p*-value (Fig 7).

The P+ responders, ISe 1387, ISe 1687, ISe 663, ISe 869 and CO7, were clustered with the genotypes that showed an intermediate response to unfertilized soil (S1 Fig). Yield-related traits such as NPT, TSE and SPC showed higher variabilities while the remaining traits such as PH, LF, LL, NC and NL exhibited lower variabilities in the analysis (S1 Fig and S2 Fig). We observed similar variations in the analysis for genotypes grown in P-fertilized soil. The most notable example was the genotype ISe 1387 on the top line of S2 Fig, and it was placed in a separate node. This genotype performed relatively well under both greenhouse and field conditions in both phosphate conditions (Figs 1A and 5 and S2 Fig). The low performers Maxima, ISe 1037 and ISe 1335 were grouped at the bottom of Fig 7 with genotypes ISe 1541 and ISe 1234, which were P+ responders for total seed yield (Fig 7).

Under greenhouse conditions and P+ treatment (S3 Fig), variability in biomass influenced the genotypic clustering pattern more than did the other traits SL, RL, RHD and RHL that showed lower variability. Under P- conditions (S4 Fig), however, biomass and SL were the major traits that diversified genotypes. Three groups in the foxtail millet genotypes were identified, under P+ condition, with the genotypes CO5, CO6, ISe 2 and Maxima forming a single cluster and are recognised as high performers under P+ (S3 Fig). Most of the low performers were grouped with genotypes that were intermediate in response to Pi conditions. Within this cluster, we could see all the high performers (CO5, CO6, ISe 2 and Maxima) under P+, which gave us confidence in the analysis. Traits measured under P- (S4 Fig) created only two groups in the foxtail millet genotypes, and SL showed higher variability. RHD and RHL showed the same variability in both P- and P+ with good responders, such as ISe 1851, CO6, ISe 1338, ISe 1305 and ISe 254, which were found to be grouped with genotypes which showed above medium response under P- (Fig 1B and S4 Fig). The genotype Maxima was clustered in a separate node, and these genotypes showed an intermediate response in P-.

## 4 Discussion

Very little information is available on intrinsic response of foxtail millet cultivars to P. So, evaluation of the P response of foxtail millet genotypes under field conditions is necessary for potential translation into agricultural practices. Field situations are complicated by environmental and edaphic factors as well as biotic influences of the co-existing flora and fauna. Moreover, root system responses and rhizosphere environment are difficult to observe and measure. Greenhouse studies, on the other hand, allow evaluation under relatively controlled conditions, and with closer observations. However, plant responses to natural conditions such as light, extreme ranges of temperature, nutrient availability and biotic interactions are not recreated under greenhouse environment. We have thus limited our greenhouse observations to root system parameters, plant biomass by dry weight and shoot length, to minimise errors. These parameters measured under greenhouse were used to complement field data to have a comprehensive evaluation of genotype performance.

In an earlier study on rice genotypes grown under natural soil and solution trials, none of the parameters was found to be significantly correlated between soil and solution experiments for relative P-use efficiency [45]. This data is a pertinent reminder that conditions in the field are very different from those in more controlled greenhouse conditions and, therefore, such data should be used as complementary to the field data to draw conclusions that are practically useful.

Further, the importance of field-based testing in two different seasons is necessary to obtain an unbiased performance of genotypes. Several factors like pH of the soil, microorganisms, presence of cations like calcium and aluminium, organic matter substances and mycorrhizal colonisation can influence the availability of Pi to the plants [36]. Genotypes that produce fewer root hairs might be compensated for by mycorrhizal colonisation in the field. A recent meta-analysis of mycorrhizal colonisation and responsiveness across a range of crop plants and wild relatives concluded that both colonisation and responsiveness vary markedly [46]. While the investigation of interaction with mycorrhizal fungi was beyond the scope of this study, it is an important consideration for future study, as is the investigation of root traits such as root branching and angle which are important traits for nutrient efficient root systems [47, 48].

Field evaluation was repeated in two different seasons involving a summer (April to July) and a monsoon (August to November) to check for consistency and reproducibility in the data. Data generated on natural field conditions in summer and monsoon seasons were correlated.

Some of the genotypes, such as ISe 1687, Maxima and ISe 1387, showed contrasting ranking patterns under field and greenhouse evaluation. Thus, it is evident that the pattern of P response in one system cannot be assumed to hold true for the response under the other. Such genotypes were considered unstable. Stable genotypes that were ranked at extreme under both field and greenhouse conditions are rare. The only exception to this from the cultivars tested was ISe 1851, which performed well under both P+ and P- conditions in the field and P- condition in the greenhouse. The results suggest that caution has to be exerted in generalising the results from greenhouse experiments to field conditions in the case of foxtail millet.

The agromorphological data obtained showed high levels of variation for P response, as expected, in the field trials. Although many genotypes gave consistent response under both seasons, a few showed variations due to environmental variations. This can well be attributed to genotypic homeostasis that varies from genotype to genotype. Moreover, this is not unexpected since the current panel of genotypes were obtained from different countries and regions [49]. This effect was more apparent with the line Maxima, belonging to the race Maxima grown in eastern China, Georgia (Eurasia), Japan and Korea. Since the variation imposed was largely due to P input, the remaining effects, either through genotypic or through genotype x environment interaction, were assumed to be uniform in both the experimental systems employed in this study. The genotypes, therefore, were ranked for growth in terms of higher values of different traits measured. The rank sums were then used as the criteria for the selection of extreme genotypes to highlight the contrast in response to P nutrition in foxtail millet genotypes. Such ranking is a simple but efficient non-parametric method in genotype selection [50, 51] but lacks statistical properties. Therefore, to ensure statistical significance between selected individuals, only those traits which showed statistical significance at 95% confidence level were used for ranking. Based on the rank sum calculated, the extreme 5% genotypes were selected. Since the traits of measurement differed under field and greenhouse screens, the rank sums were computed separately. This strategy helped identified extreme genotypes under two systems and under two levels of P nutrition.

Under Pi insufficiency conditions, plants reallocate resources to roots, increasing the root-shoot ratio. Increase in RHD and RHL helps to improve foraging for P in the topsoil because more P is available in this layer due to the presence of moisture and organic matter, increased microbial activity and oxygen availability [12, 52]. The formation of root hairs has been considered one of the key strategies to overcome Pi starvation response with minimal carbon cost [53]. According to the previous studies, foxtail millet produces a larger root system in terms of crown root length and lateral root number, length, and density under Pi deficiency [54]. Under low nitrogen stress, foxtail millet produces much higher specific root length and increased root diameter [55]. However, this is the first report analyzing the RHD and RHL in response to low Pi stress in foxtail millet. Root proliferation in soil as a response to low P [52] has been reported in rice conditioned by the QTL, *Pup1* [26, 56]. In *Arabidopsis*, increase in number and length of root hairs improved Pi uptake under Pi-limiting conditions [57]. So, root traits are considered important targets for breeding for better nutrient acquisition as well as for enhancing photosynthesis and plant productivity [58–60]. Ahmad et al. [61] have reported that root expansion, density, and length of foxtail millet genotypes Yugu1 has been increased under low P due to carbon provision to the root system that significantly increased the Pi uptake in the soil. Our results show that it is not only the root hair density and root hair length that are the deciding factors. The efficiency and density of the proteins that transport phosphate from the soil into the roots may also play a role. Initial investigations on the yield and expression of PHT1 family phosphate transporters in seven millets show wide variation between millets [20]. Future studies on the variation within the various foxtail millet genotypes may help us shift focus from gross features such as RHL and DHL to the genes, making targeted breeding easier and faster.

P within the plant is translocated from the roots to the shoot. The genotypes that possess an active sink of P are P-use efficient. Therefore, the P content of the extreme genotypes was investigated by assaying total and inorganic P contents in root and shoot tissues grown under both greenhouse and field conditions. There was good agreement between plant P content under field and greenhouse conditions indicating that the P response pattern of the genotypes was consistent and stable. However, the relationship did not hold for extreme genotypic groups, because of the high magnitude of specific P responses in these sets of genotypes. P content was high in plants when P was applied (P+) in both the systems, because of higher availability in the growing substrate. P uptake efficient genotypes can efficiently harness P under P+ conditions. Furthermore, good responders under P+ and P- conditions accumulated more P in the plant system than the poor responders. Those with high P in the plant system also have better utilisation efficiency if they can effectively transport P between different utilization points of growth and metabolism. Hence, in this experiment, good responders under P- can be construed as low P tolerant genotypes, while good responders under P+ can be identified as P loving genotypes. Low P tolerant genotypes with good seed yield such as ISe 663 and ISe 869 are highly preferred for future breeding programmes in foxtail millet targeting P starvation tolerance. Moreover, the wide genetic variation for P responses, observed in this study, signal the possibility of the genetic improvement of foxtail millet for P use efficiency as seen in other cereal crops such as rice [62], wheat [63], barley [58] and maize [48].

The changes in growth, biomass and yield have been recognized as important indicators of Pi deficiency tolerance as reported in other cereals including oat [24], rice [25, 26], maize [27] and sorghum [28]. Higher biomass in genotypes that produced more, and longer root hairs is an indication of better P utilization. However, having a high number of root hairs per root was not sufficient. Genotype ISe 1387 had good root hairs under P- conditions but produced extremely small biomass in the greenhouse experiment. However, it was the best performer in the field experiment. Other factors such as efficient symbioses or P acquisition ability from organic sources may influence its potential biomass and affect efficient Pi uptake from the soil. Some foxtail genotypes had very little or minute root hairs produced under P+ conditions, which suggested that proliferation of root hairs was a low P response. In barley, a root hairless mutant reduced phosphate uptake under the P- condition and was also associated with decreased biomass production [64, 65].

The good responders under P- condition in the study had both high RHD and RHL. However, in some crops, such as soybean, RHD and RHL under low P conditions showed a negative association [66]. This could be considered as an adaptive response in foxtail millet, a cereal with an adventitious root system, as against the tap root system of soybean wherein a trade-off in terms of carbon use efficiency has been maintained, because combining both RHL and RHD will be too costly in terms of carbon usage. However, in common bean, both higher RHL and RHD were found in P-efficient genotypes [67].

Studies of pearl millet and sorghum showed enormous genetic variation for PUtE in natural field conditions based on their responses under P starvation [68, 69]. In our field-based study, the high responding genotypes under unfertilized conditions did almost as well as the top performers under P-fertilized conditions, suggesting high phosphate acquisition and/or use efficiency of these genotypes.

Some genotypes such as ISe 1234, ISe 1736, CO-7 and Maxima have smaller inflorescence under P+ (Fig 5). This suggests that the excess of P may not be good for some genotypes. The optimum amount of P to maximise the yield may vary from genotype to genotype in foxtail millet.

The reduced size of the inflorescence does not translate to reduced seed yield in all genotypes. For example, in spite of smaller inflorescence, the seed yield of ISe 1234 was significantly higher under P+. Moreover, in spite of increased size of inflorescence, ISe 663, ISe 869, ISe 1037 and ISe 1338 showed marginal reduction in seed yield. This suggests that the inflorescence size alone is not a good indicator of seed yield in foxtail millet genotypes. P availability has an impact on the NC/panicle, SPC, TSE /panicle. The response to excess of P could also be the differential response in terms of RHL and RHD. Future studies may help us understand the molecular dynamics of the differential responses of these traits under P fertilisation.

Seed yield is the ultimate target of any crop-based study. Plasticity analysis of P response was therefore limited to total seed yield trait. Plasticity is the difference between the P+ and P- response of any genotype under a given cultural condition expressed as the percentage over the average of that genotype in both conditions. Since the response under low P is subtracted from that under high P, positive plasticity indicates better performance under fertilized conditions and vice-versa. The genotypes ISe 1387 and ISe 1687 performed well in the natural field in P-fertilized and unfertilized conditions. However, both are P+ responders for TSE, based on plasticity analysis, because they were more productive under fertilized condition. The plasticity of these genotypes is low and closer to the axis (19.3% and 6.6% respectively), which indicated that they could perform equally well under low as well as high P conditions, irrespective of the magnitude of yield, with a slight edge towards high P response. Such genotypes are highly preferable, as they can be grown under varying P situations. While there was no broad correlation between greenhouse and field performance, some genotypes did do well under both conditions. These included the local varieties CO5 and CO7. These results indicate that local varieties are well adapted to the conditions and it would be interesting to compare the responses of the local varieties with those of the cultivar Maxima at the molecular and biochemical level and to investigate their phosphate transporters to gain more insights into the mechanisms behind these distinct responses.

Clustering analysis and representation of the data as heat maps allowed an overview of the performance of all genotypes under both greenhouse and field conditions. Performances in the greenhouse and in the field were not well correlated in the clusters, and no convincing correlation between any greenhouse measured parameter and seed yield in unfertilized conditions could be ascertained. However, seed yield under P-fertilized and unfertilized conditions was correlated, which suggested that genetic control of seed yield was largely independent of P supply.

The total P and Pi contents of high-responding genotypes were much higher than those of low responding genotypes, under both P-fertilized and unfertilized fields. Although all the genotypes grown in the natural field were able to grow in unfertilized soil, their uptake efficiency varied considerably resulting in growth differences. This was reflected in the contrast between good and poor responding genotypes.

## 5 Conclusion

We examined the level of variation seen among 54 foxtail millet genotypes for P response in both greenhouse and natural field conditions with a focus on low P stress tolerance. The selection based on rank sums could effectively identify extreme genotypes under P+ and P- conditions with a contrasting response. We could identify genotypes that were low P tolerant and high P responding and several of them with intermediate behaviour.

This study provides comprehensive information about low P response of foxtail millet genotypes, which is conspicuously lacking in the literature. This may lay the foundation for further breeding and molecular studies to improve the foxtail millet for PUE.

Millets are small-seeded cereals in the family Poaceae, mainly cultivated and consumed by people in the tropics. Millet grains are rich in essential nutrients such as calcium, magnesium, zinc and iron and form a major source of nutrition and food security for millions in less developed nations [70]. *Setaria italica* and its wild relative, green foxtail (*Setaria viridis*) are considered good models for nutrient management in other millets [71, 72]. Foxtail millet is also closely related to bio-fuel grasses such as switchgrass and Napier grass [73, 74]. Hence, we suggest that the results presented here have implications in understanding the response of other important crops.

## Supporting information

S1 Table Details of foxtail millet genotypes used in the present study. A total number of 26 genotypes originating in India, out of which three were locally cultivated varieties. Remaining 28 genotypes that have been studied from other countries, representing a wide geographic coverage.
(DOCX)

S2 Table The raw data, the difference in mean values and plasticity analysis between Pi fertilized (P+) and unfertilized (P-) treatments of seedlings grown in natural field condition. (XLSX)

S3 Table The difference in mean values between P+ (300 μM Pi) and P- (10 μM Pi) treatments of seedlings grown in greenhouse condition. (DOCX)

S4 Table Details on R scripts used for creating figures. (DOCX)

S1 Fig A clustered heatmap for 54 genotypes of foxtail millet response under unfertilized (P) soil. Genotypes are scaled and hierarchically clustered by Euclidean distance. Yellow equates to a high value and red to a low value. PH, plant height; NPT, productive tiller number; NL, leaf number; LL, leaf length; LF, flower length; NC, cluster number; SPC, seeds per cluster; TSE total seed yield.
(TIF)

S2 Fig A clustered heatmap for 54 genotypes of foxtail millet response under Pi fertilized (P+) soil. Genotypes are scaled and hierarchically clustered by Euclidean distance. Yellow equates to a high value and red to a low value. PH, plant height; NPT, productive tiller number; NL, leaf number; LL, leaf length; LF, flower length; NC, cluster number; SPC, seeds per cluster; TSE total seed yield.
(TIF)

S3 Fig A clustered heatmap for 54 genotypes of foxtail millet response to P+ (300 μM Pi) under greenhouse conditions. Genotypes are scaled and hierarchically clustered by Euclidean distance. Yellow equates to a high value and red to a low value. SL, shoot length; RL, root length; RHD, root hair density; RHL, root hair length.
(TIF)

S4 Fig A clustered heatmap for 54 genotypes of foxtail millet response to P- (10 μM Pi) under greenhouse conditions. Genotypes are scaled and hierarchically clustered by Euclidean distances. Yellow represents a high value and red a low value. SL, shoot length; RL, root length; RHD, root hair density; RHL, root hair length.
(TIF)
