# Supplementary material for: Phenotypic responses of foxtail millet (Setaria italica) genotypes to phosphate supply under greenhouse and natural field conditions
Source: PLoS One. 2020 Jun 3;15(6):e0233896. doi: 10.1371/journal.pone.0233896 (PMC7269269; doi:10.1371/journal.pone.0233896)
Supplement: S1 Table — A total number of 26 genotypes originating in India, out of which three were locally cultivated varieties. Remaining 28 genotypes that have been studied from other countries, representing a wide geographic coverage. (DOCX) [file pone.0233896.s001.docx]

**Table S1.** Details of foxtail millet genotypes used in the present study. A total number of 26 genotypes originating in India, out of which three were locally cultivated varieties. Remaining 28 genotypes that have been studied from other countries, representing a wide geographic coverage.

| **S. No** | **Name of the genotype** | **Alternate Name/ Number** | **Origin** |
| --- | --- | --- | --- |
| 1 | ISe 2 | Korra | India |
| 2 | ISe 96 | Bhedi | India |
| 3 | ISe 132 | Kangani | India |
| 4 | ISe 254 | Mobbu navne | India |
| 5 | ISe 375 | Koni dhan | India |
| 6 | ISe 376 | - | India |
| 7 | ISe 388 | Tangum | India |
| 8 | ISe 480 | Var.hungsheku | China |
| 9 | ISe 507 | M 235; B 35 | Kenya |
| 10 | ISe 663 | FAO ≠ 11324 | Switzerland |
| 11 | ISe 710 | - | India |
| 12 | ISe 719 | - | Pakistan |
| 13 | ISe 745 | - | India |
| 14 | ISe 748 | - | India |
| 15 | ISe 751 | - | India |
| 16 | ISe 783 | - | India |
| 17 | ISe 869 | Rgot | India |
| 18 | ISe 907 | SE 201 | India |
| 19 | ISe 909 | SE 480 | India |
| 20 | ISe 963 | SE 3045 | India |
| 21 | ISe 995 | SE 7230/3-1 | India |
| 22 | ISe 1037 | IPM 1626 | Lebanon |
| 23 | ISe 1151 | NESE 82: 3876-1 | Syria |
| 24 | ISe 1161 | NESE 87-2: 3881-2 | Syrian Arab Republic |
| 25 | ISe 1181 | EC 130490 | China |
| 26 | ISe 1209 | EC 131210: WIR 864 | Russia & CISs |
| 27 | ISe 1234 | EC 131236: WIR 1030 | Russia & CISs |
| 28 | ISe 1254 | EC 131256: WIR 1346 | Russia & CISs |
| 29 | ISe 1269 | EC 134287 | South Africa |
| 30 | ISe 1299 | EC 134283; PI 250025 | Iran |
| 31 | ISe 1302 | EC 134286; Pi 207502 | Afghanistan |
| 32 | ISe 1305 | PI 283988; EC 134289 | Spain |
| 33 | ISe 1320 | EC 134329; PI 363068 | USA |
| 34 | ISe 1335 | EC 134320; PI 290461 | Hungary |
| 35 | ISe 1338 | EC 134257; PI 173805 | Turkey |
| 36 | ISe 1354 | - | India |
| 37 | ISe 1387 | EC 135535 | Sri Lanka |
| 38 | ISe 1468 | SIA 326 | India |
| 39 | ISe 1474 | EC 155192-1 | United Kingdom |
| 40 | ISe 1541 | T 132/12 | India |
| 41 | ISe 1563 | Wooljin 7 | Republic of Korea |
| 42 | ISe 1610 | - | Malawi |
| 43 | ISe 1655 | 40051 | Taiwan |
| 44 | ISe 1687 | Jhum | India |
| 45 | ISe 1736 | Acc No. 6542 | Nepal |
| 46 | ISe 1745 | Shwe sutkon | Myanmar |
| 47 | ISe 1820 | ISe 231 A; Wari | India |
| 48 | ISe 1851 | ISe 275 A: Kangani | India |
| 49 | ISe 1888 | ISe 410 B; Roll boer mann | Ethiopia |
| 50 | ISe 1892 | ISe 472 A | USA |
| 51 | CO-5 | - | India |
| 52 | CO-6 | - | India |
| 53 | CO-7 | - | India |
| 54 | Maxima | Bs 3875 | - |
