# Supplementary material for: Phenotypic responses of foxtail millet (Setaria italica) genotypes to phosphate supply under greenhouse and natural field conditions
Source: PLoS One. 2020 Jun 3;15(6):e0233896. doi: 10.1371/journal.pone.0233896 (PMC7269269; doi:10.1371/journal.pone.0233896)
Supplement: S4 Table — (DOCX) [file pone.0233896.s004.docx]

**Table S4.** Details on R Scripts used for creating figures

| **R script used to generate Fig 7** | **R script used to generate supplementary figures S1, S2, S3 and S4** |
| --- | --- |
| library (pvclust)  data <- read.table(“data.txt”)  result <- pvclust(data, nboot=300)  plot(result) | library(gplots)  Y <- read.delim("D:/Desktop/Y.txt", row.names=1)  y<-as.matrix(Y)  ynorm<-scale(y)  heatmap.2(ynorm) |
