# Supplementary figures and images for: Phenotypic responses of foxtail millet (Setaria italica) genotypes to phosphate supply under greenhouse and natural field conditions
Source: PLoS One. 2020 Jun 3;15(6):e0233896. doi: 10.1371/journal.pone.0233896 (PMC7269269; doi:10.1371/journal.pone.0233896)

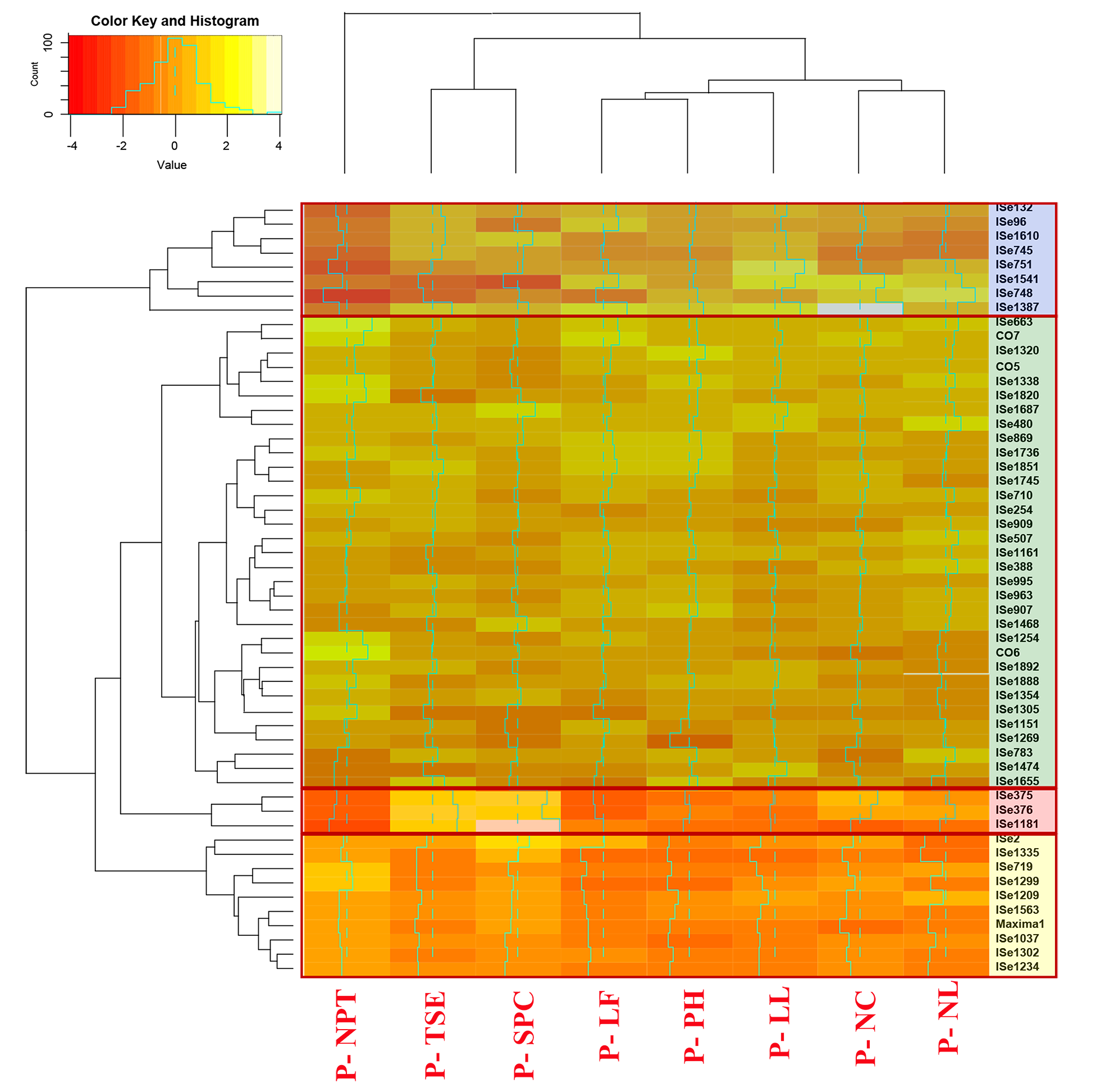

Supplement: S1 Fig — Genotypes are scaled and hierarchically clustered by Euclidean distance. Yellow equates to a high value and red to a low value. PH, plant height; NPT, productive tiller number; NL, leaf number; LL, leaf length; LF, flower length; NC, cluster number; SPC, seeds per cluster; TSE total seed yield. (TIF) [file pone.0233896.s005.tif]

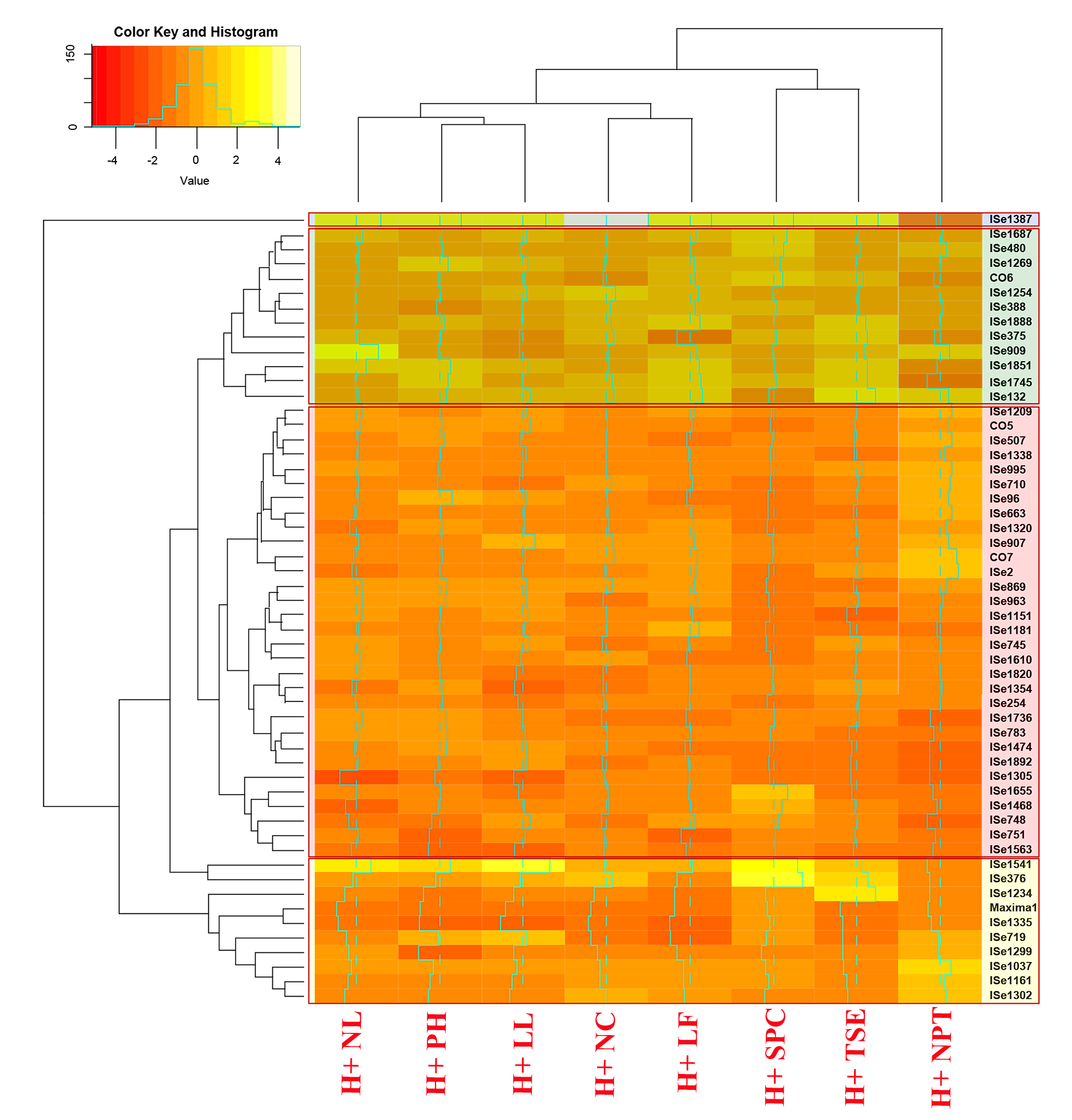

Supplement: S2 Fig — Genotypes are scaled and hierarchically clustered by Euclidean distance. Yellow equates to a high value and red to a low value. PH, plant height; NPT, productive tiller number; NL, leaf number; LL, leaf length; LF, flower length; NC, cluster number; SPC, seeds per cluster; TSE total seed yield. (TIF) [file pone.0233896.s006.tif]

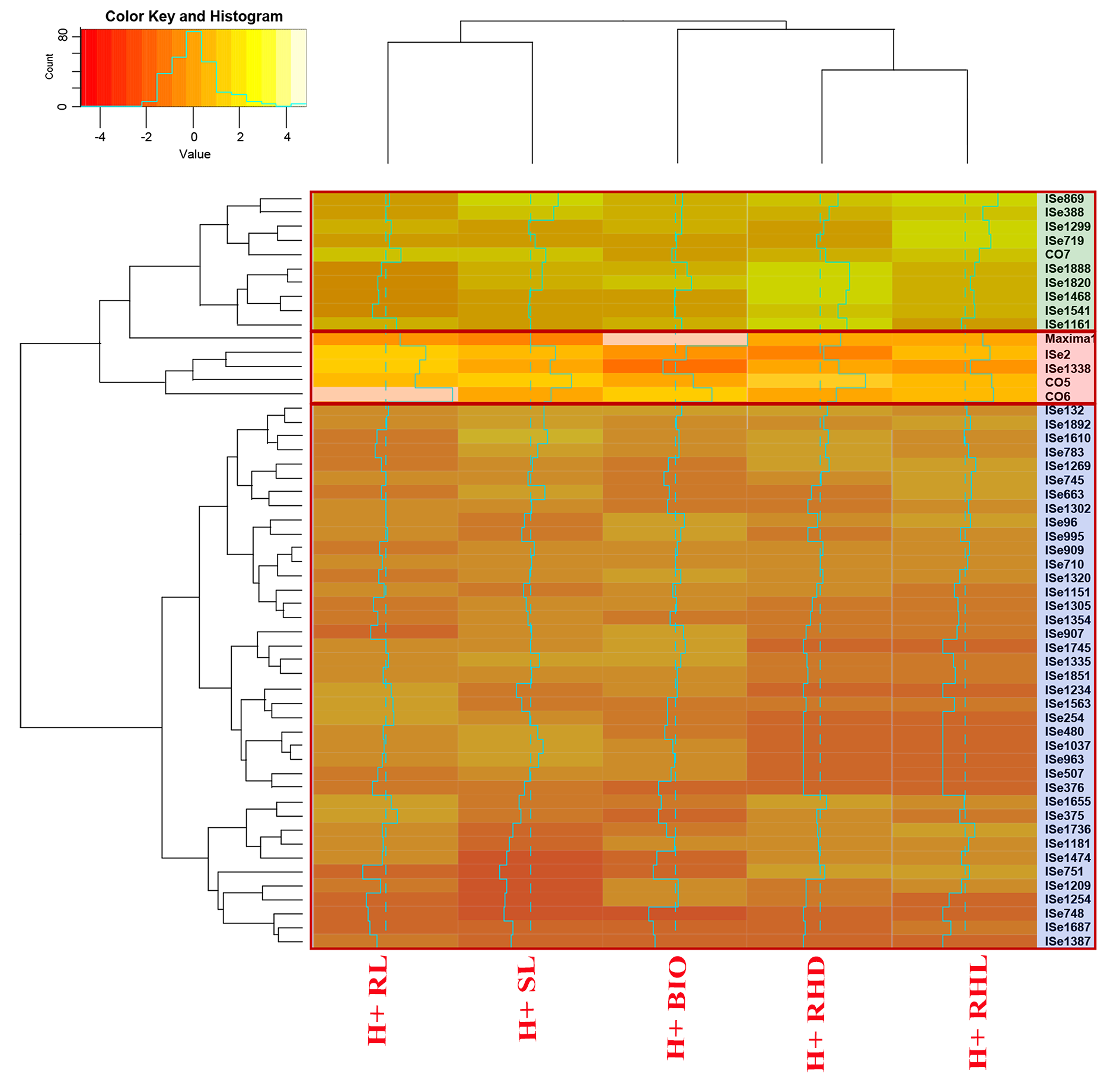

Supplement: S3 Fig — Genotypes are scaled and hierarchically clustered by Euclidean distance. Yellow equates to a high value and red to a low value. SL, shoot length; RL, root length; RHD, root hair density; RHL, root hair length. (TIF) [file pone.0233896.s007.tif]

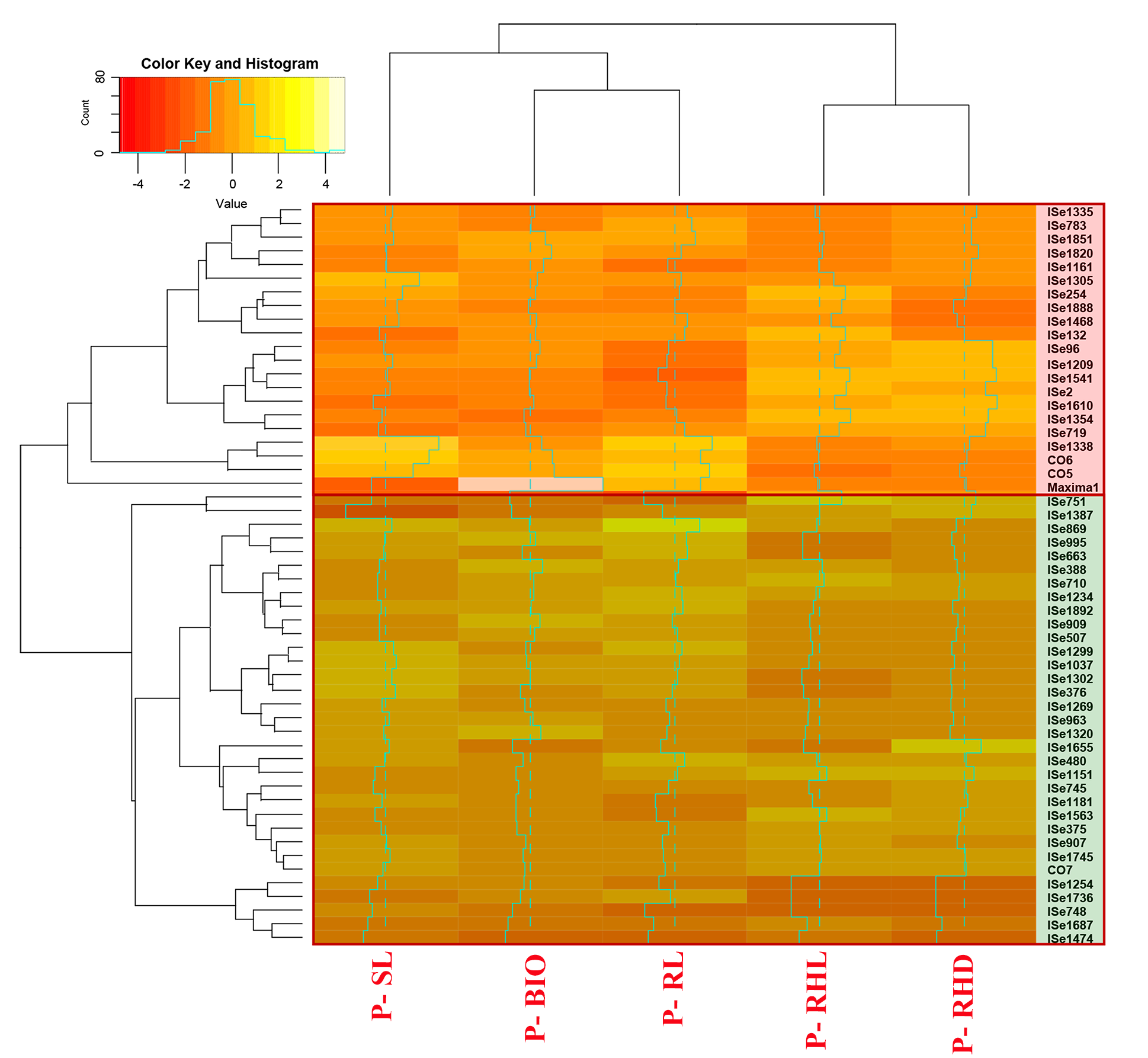

Supplement: S4 Fig — Genotypes are scaled and hierarchically clustered by Euclidean distances. Yellow represents a high value and red a low value. SL, shoot length; RL, root length; RHD, root hair density; RHL, root hair length. (TIF) [file pone.0233896.s008.tif]
